# Convective and diffusive effects on particle transport in asymmetric periodic capillaries

**DOI:** 10.1371/journal.pone.0183127

**Published:** 2017-08-25

**Authors:** Nazmul Islam, Stanley J. Miklavcic, Bronwyn H. Bradshaw-Hajek, Lee R. White

**Affiliations:** 1 Phenomics and Bioinformatics Research Centre, University of South Australia, Mawson Lakes, South Australia, Australia; 2 Mathematics Discipline, Science, Engineering and Technology School, Khulna University, Khulna, Bangladesh; North China Electric Power University, CHINA

## Abstract

We present here results of a theoretical investigation of particle transport in *longitudinally asymmetric* but *axially symmetric* capillaries, allowing for the influence of both diffusion and convection. In this study we have focused attention primarily on characterizing the influence of tube geometry and applied hydraulic pressure on the magnitude, direction and rate of transport of particles in axi-symmetric, saw-tooth shaped tubes. Three initial value problems are considered. The first involves the evolution of a fixed number of particles initially confined to a central wave-section. The second involves the evolution of the same initial state but including an ongoing production of particles in the central wave-section. The third involves the evolution of particles a fully laden tube. Based on a physical model of convective-diffusive transport, assuming an underlying oscillatory fluid velocity field that is unaffected by the presence of the particles, we find that transport rates and even net transport directions depend critically on the design specifics, such as tube geometry, flow rate, initial particle configuration and whether or not particles are continuously introduced. The second transient scenario is qualitatively independent of the details of how particles are generated. In the third scenario there is no net transport. As the study is fundamental in nature, our findings could engender greater understanding of practical systems.

## Introduction

In a recent paper [1] we investigated laminar hydrodynamic flow through infinite axi-symmetric, periodic capillaries by means of a boundary element method. The flow field, even while laminar, exhibited vortex behavior in certain regions of the undulating tube, whose geometry was characterized by a narrow throat of specified radius and length, and a finite-length expansion zone, whose radius was a function of the axial coordinate. We quantified the onset of flow recirculation appearing in the expansion sections of the tube as a function of throat and expansion zone dimensions, focusing the study on tube geometries that possessed axial symmetry. We found that for a given geometric shape, a critical expansion zone dimension existed above which a flow vortex region developed and grew (in each repeat section of the periodic tube). The growth as a function of geometric parameter was limited by the appearance of a second recirculation zone above the first.

The natural question we now ask is what influences, if any, do these recirculation zones have on the net transport of particles suspended in the fluid. Assuming that the particles do not alter the makeup of the flow field, we are specifically led to ask the question of whether there is an interplay between flow recirculation and particle diffusion. Is recirculation in combination with or in competition with diffusion, in the net transport of particles? To the best of our knowledge this question has not been addressed. The study presented here may provide some elemental physical understanding to achieve the goal of protein, DNA or other macromolecular separation, separation of biological cells, as well as fine mineral particle separation [2–7] and their transport. There are many features of the current work which point to it complementing the recent analysis on hydrodynamic particle transport in tubes by Herringer *et al.* [8]. Considering a more direct application, the model and simulation results can facilitate greater understanding of subcutaneous drug delivery. Drug molecules are introduced to a vascular system either by direct infusion (injection) into a blood vessel or at the conclusion of a diffusive process through extra-vascular tissue following topical (skin) application. The *in vivo* transport is then dictated by vascular diffusion from the point of entry and influenced by convective forces due to the action of the heart pump [9–14]. Although a direct comparison between our model results and practical measurements in living tissues is not realistic, some useful insight into the latter system may be derived.

We thus investigate the dynamic behavior of particle distributions in infinite, periodic tubes filled with a viscous liquid, which is disturbed by the action of a periodic pressure field that drives the fluid forwards and backwards with no net fluid flow. In this theoretical study three scenarios are considered. First consideration is given to the initial value problem of a fixed number of particles, initially distributed over one wave-section, and then allowed to convect and diffuse into adjoining wave-sections. Second is an initial value problem with the same initial state but to which is added a continual supply of particles in the same single wave-section. Both scenarios represent transient states of the system and arguably have a correspondence with localized drug introduction in a blood vessel (see, *e.g.*, Fig 4 in Dancik *et al.* [13]). Finally, we consider an initial value problem adopting the state of a completely filled tube, with particles subjected to an ongoing oscillatory fluid flux. In the latter case the entire system is spatially periodic. The dynamics of these systems is governed by a hydrodynamic set of equations for the fluid flow and a diffusive-convective equation with a forcing of particles due to the motion of the suspending fluid. In the second scenario, a particle generation term, applied in one section only, appears in the diffusive-convective equation. The study, as a function of tube geometry, is fundamental in that the questions posed above are answered by monitoring the net direction and rate of transport of particles through the tube at subsequent times.

We consider tube shape and geometry as primary factors influencing the transport phenomenon. We establish the effect on transport of longitudinal asymmetry, amplitude and wavelength of corrugation, sharpness of expansion regions as well as length and radius of the throat regions. We concentrate on geometric variants of a smoothed saw-tooth profile. That is, in contrast to the study reported by Islam, et al. [1], we focus on tube profile *asymmetry*. The theoretical model is described in the next section, while in the Simulation Results section we explore a sufficient extent of parameter space to characterize the contributions of diffusion and convection as well as the effect of geometry on facilitating particle transport in the three scenarios. A discussion of the phenomenon of particle transport within an asymmetric, periodic capillary, based on our findings, is relegated to the Discussion section.

## Theoretical model and governing equations

We consider a dispersion of particles of number density $\bar{c}\left(\bar{x},\bar{t}\right)$ suspended in an incompressible fluid of density *ρ* and dynamic viscosity *μ* confined to an infinite, periodic axi-symmetric capillary. Gravitational effects are ignored, which is equivalent to assuming that the particle density is equal to the density of the fluid. Many of the fluid and particle assumptions outlined below are similar to the assumptions adopted in recent work on peristaltic transport of nanoparticles in micro-channels [15, 16] (note that the latter work involves a two-dimensional system in contrast to our axi-symmetric system).

**Fig 1:**
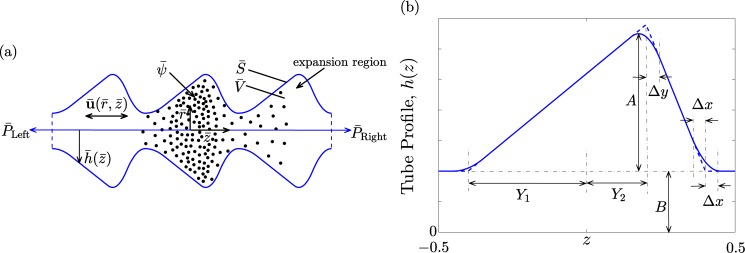
(a) Schematic of an infinitely long, periodic tube of longitudinally asymmetric profile exhibiting particles introduced ($\bar{\psi }$) in the central wave-section and migrating out under diffusion and convection. (b) Detailed schematic of an axi-symmetric, smoothed, saw-tooth tube. Non-dimensional geometric construction particulars are indicated in the figure. The leading edge of the saw-tooth profile is that part in the positive *z* half of the wave-section having the steeper sloped boundary. The trailing edge is the shallower sloped boundary.

A point on the surface of the axi-symmetric tube is given by the vector position $\bar{y}=\bar{z}\hat{z}+\bar{h}\left(\bar{z}\right)\hat{r}$, where $\hat{z}$ and $\hat{r}$ are unit vectors in the longitudinal and radial directions, respectively, and $\bar{h}\left(\bar{z}\right)$ defines the tube surface. By assumption, spatial periodicity implies that $\bar{h}\left(\bar{z}\right)=\bar{h}\left(\bar{z}+L\right)$ where *L* is the spatial period of the periodic profile. The geometric variables implicit in $\bar{h}$ will depend on the shape assumed. However, characteristic to all shapes that we consider is a throat region of radius, $\bar{B}$, and an expansion region of maximum radius, $\bar{A}+\bar{B}$. The shape we consider predominantly in this paper is that of an asymmetric saw-tooth profile. However, for comparison we also consider the special cases of a symmetric triangular profile and a straight cylinder. Both the saw-tooth and triangular profiles have been smoothed to eliminate corners. Fortunately, this smoothing also appears in experimental studies. The profiles we study then have a differentiable surface tangent vector. A schematic of the periodic tube in longitudinal section and an illustration of the smoothed saw-tooth profile with defining parameters are shown in Fig 1. In the following sections we refer to “leading” and “trailing” edges of the saw tooth profile as explained in the figure.

The suspending fluid is assumed to be in a state of flow, driven by a time-varying pressure gradient $\Delta \bar{P}\left(\bar{t}\right)/L$, where $\Delta \bar{P}\left(\bar{t}\right)$ is the instantaneous pressure drop across one wave-section. We shall assume both low Reynolds number flow and a sufficiently slow time variation of the pressure to neglect the transient and convective terms in the Navier-Stokes equations and allow use of the time independent Stokes system of equations. The latter assumption implies that relative to the time scale of the pressure variation, the flow is able to adjust immediately. We also assume that the flow is instantaneously responsive to the slowly varying applied pressure so that the time dependence of the fluid velocity mimics that of the applied pressure.

We also assume that the particle dispersion is sufficiently dilute and particle size sufficiently small that one can neglect particle-particle and particle-surface interactions. More importantly, as mentioned earlier, with these assumptions we neglect the influence of the particles themselves on the development of the fluid flow field. Under these assumptions, the hydrodynamic and particle transport problems are partially decoupled.

### Hydrodynamic flow in an infinite periodic tube

Given the assumptions outlined above, the hydrodynamic problem is decoupled from the particle transport problem. The former problem can then be solved within a single wave-section of the tube under the condition of periodicity. This is in fact the problem solved by Islam, et al. [1] by a boundary element method applicable to an infinite periodic tube. The reader is referred to that work for details. In summary, the governing equations are the time-independent, linear momentum and continuity equations, respectively,

$$\begin{array}{c} {\bar{\nabla }}^{2}\bar{u}=\frac{1}{\mu }\bar{\nabla }\bar{p}\;,\;\;\bar{\nabla }\cdot \bar{u}=0, \end{array}$$ (1)

with a stick boundary condition on the tube surface, $\bar{S}$,

$$\begin{array}{c} \bar{u}\left(\bar{x}\right)=0,\;\;\bar{x}\in \bar{S}, \end{array}$$ (2)

and pressure difference $\Delta \bar{P}=\bar{P}\left(\bar{x}\right)-\bar{P}\left(\bar{x}+L\hat{z}\right)$ applied across one wave-section of the tube. For flow in an infinite periodic tube one can write $\bar{P}\left(\bar{x}\right)=-{\left(\Delta P\right)}_{amp}\bar{z}/L+\bar{℘}\left(\bar{x}\right)$ where the amplitude (Δ*P*)_*amp*_ will later be replaced by a slowly varying function of time and where, meanwhile, $\bar{℘}\left(\bar{x}\right)=\bar{℘}\left(\bar{x}+L\hat{z}\right)$ is a periodic function of $\bar{z}$. In Eq (1), commonly referred to as the Stokes system, $\bar{p}\left(\bar{r},\bar{z}\right)$ is the hydrodynamic pressure, *μ* is the viscosity of the fluid and $\bar{u}\left(\bar{z},\bar{r}\right)$ is the flow velocity.

Presuming comparable radial and axial characteristic length scales, in non-dimensional variables, $z=\bar{z}/L,\;r=\bar{r}/L,\;p=\bar{p}/{\left(\Delta P\right)}_{amp},\;u=\bar{u}\mu /\left(L{\left(\Delta P\right)}_{amp}\right)$, the Stokes equations become

$$\begin{array}{c} \nabla^{2}u=\nabla p\;,\;\;\nabla \cdot u=0. \end{array}$$ (3)

The above spatial non-dimensionalisation would not be applicable for channels with highly disparate lateral and longitudinal characteristic lengths for which case the *z* and *r* variables should be scaled differently (see, *e.g.*, [15, 16]). In Islam et al. [1] we expressed the fundamental solution of this system in boundary integral form,

$$\begin{array}{ccc} u(x) & = & \frac{1}{4\pi }\int_{S}^{}dS\left(y\right)\;G(x,y)\cdot F(y)\\ & & \;-\frac{1}{4\pi }\int_{S}^{}dS\left(y\right)\;H(x,y)\cdot u(y),\;\;\;\;x\in S\;, \end{array}$$ (4)

$$\begin{array}{ccc} u(x) & = & \frac{1}{8\pi }\int_{S}^{}dS\left(y\right)\;G(x,y)\cdot F(y)\\ & & \;-\frac{1}{8\pi }\int_{S}^{}dS\left(y\right)\;H(x,y)\cdot u(y),\;\;\;\;x\in V, \end{array}$$ (5)

evaluated on the tube surface and interior, respectively. Here, *dS* is an element of surface area on the boundary *S* at **y**, *V* is the interior tube domain and $F\left(y\right)=-\Sigma \left(y\right)\cdot \hat{n}\left(y\right)$ is the force per unit area exerted on the fluid by the boundary at position **y** (boundary force), with Σ(**y**) as the stress tensor,

$$\begin{array}{c} \Sigma_{\text{ij}}=-p\;\delta_{\text{ij}}+(\frac{\partial u_{i}}{\partial x_{j}}+\frac{\partial u_{j}}{\partial x_{i}}). \end{array}$$

The surface normal $\hat{n}\left(y\right)=\left({\hat{n}}_{r},{\hat{n}}_{z}\right)$ is directed outward from the volume *V*. Also, **G**(**x**, **y**) and **H**(**x**, **y**) are known functions of the sample point **x** and source point **y**, defined as follows [17]:

$$\begin{array}{c} G_{\text{ij}}\left(x,y\right)=\frac{\delta_{\text{ij}}}{\epsilon }+\frac{{\hat{x}}_{i}{\hat{x}}_{j}}{\epsilon^{3}}\;\text{and}\;H_{\text{ij}}\left(x,y\right)=-6\frac{{\hat{x}}_{i}{\hat{x}}_{j}{\hat{x}}_{k}}{\epsilon^{5}}n_{k}. \end{array}$$

where $\epsilon =|\hat{x}|$ and $\hat{x}=y-x$.

Eqs (4) and (5) can be simplified considerably for an infinite, axi-symmetric and periodic tube. Axi-symmetry obviates the dependence on azimuthal angle. Consequently, integration over this variable can be performed immediately, reducing the two-dimensional surface integrals effectively to one-dimensional integrals over the boundary line defined by a single longitudinal section. Periodicity can be utilized to reduce these integrals over the infinite tube to a set of finite, one-dimensional integrals confined to one wave-section. Details of this procedure can be found in Islam et al. [1].

### Axisymmetric particle diffusive-convective transport

We consider the scenario of time and spatial scales that lead to diffusive effects that are commensurate with the influence of fluid convection. We thus consider here a macroscopic, continuum description and solve the axi-symmetric convective diffusion equation for the particle number density, $\bar{c}\left(\bar{r},\bar{z},\bar{t}\right)$:

$$\begin{array}{c} \frac{\partial \bar{c}}{\partial \bar{t}}=-\bar{\nabla }\cdot \{-\bar{\nabla }(D_{th}\bar{c})+\bar{c}\bar{u}\}+\bar{\psi }, \end{array}$$ (6)

where particle size, *a*, is partially and indirectly taken into account through its appearance in the Stokes-Einstein formula *D*_*th*_ = *k*_*B*_*T*_*temp*_/(6*πμa*) for the diffusion constant, where *k*_*B*_ is Boltzmann’s constant and *T*_*temp*_ is temperature in degrees Kelvin. This equation governs the distribution of point particles assuming a fluid convection contribution that is driven by a fluid velocity determined in the absence of particles, *i.e.*, $\bar{u}\left(\bar{r},\bar{z},\bar{t}\right)$ is treated as known in this equation.

The final term in Eq (6), $\bar{\psi }\left(\bar{z},\bar{t}\right)$, which *only* appears in the second initial value problem considered here, is a particle generation term that is nonzero only in the central wave-section, $-L/2\leq \bar{z}\leq L/2$ (details are given later). It represents the uniform (in $\bar{r}$) and continual (in $\bar{t}$) production within that wave-section of particles that are subsequently transported into neighboring sections. This term has been added with little consideration for how it could be engineered in practice although one can imagine it to model approximately the introduction of particles (under adequate pressure) through a porous central section boundary. Maintaining an ongoing generation term fortuitously also ensures that derivative calculations remain significantly greater than numerical error by one to two orders of magnitude, or greater. Given that we are interested in transport through an infinite periodic, axi-symmetric capillary of longitudinal cross section, $\bar{h}\left(\bar{z}\right)$, it suffices to consider particles introduced by means of a smooth function that possesses the properties of (a) being non-zero only within the central wave-section, *W*_0_, approaching zero smoothly as $\bar{z}\to \pm L/2$, (b) being a differentiable function of $\bar{z}$, and (c) resulting in a zero first moment (see Simulation results) when integrated over the length of the central wave-section. A function satisfying these conditions is

$$\begin{array}{c} \bar{\psi }\left(\bar{z},\bar{t}\right)=\{\begin{array}{cc} \frac{\Psi \left(\bar{t}\right){\bar{h}}_{min}^{2}}{\bar{h}{\left(\bar{z}\right)}^{2}}[\cos(\frac{2\pi \bar{z}}{L})+1], & \bar{t}\geq 0,\bar{x}\in W_{0},\\ 0, & \text{otherwise}, \end{array} \end{array}$$ (7)

where *h*_*min*_ = *B*,

$$W_{0}=\{\left(\bar{r},\bar{z}\right);\bar{r}\in \left(0,\bar{h}\left(\bar{z}\right)\right),-L/2\leq \bar{z}\leq L/2\}$$

and Ψ(0) = *c*_0_, Ψ(*t*) = *c*_0_/*T* for $\bar{t}>0$ and *c*_0_ is a prescribed scalar. This assumes a constant (in $\bar{t}$) and uniform (in $\bar{r}$) supply of particles in *W*_0_; over a period of the pressure oscillation, *T*, the function $\bar{\psi }\left(\bar{z},\bar{t}\right)$ generates a constant particle number, $\pi c_{0}L{\bar{h}}_{min}^{2}$. Including Eqs (7) in (6) allows particles to be introduced without preventing movement across *W*_0_.

Having thus described this functional form and associated initial state, we demonstrate in S1 Appendix (Section C) that qualitative behavior of this initial value problem remains unchanged if we instead invoke a simpler and cruder initial state, $c\left(\bar{r},\bar{z},0\right)=c_{0}$ for $\bar{x}\in W_{0}$, 0 otherwise, and a different functional form,

$$\begin{array}{c} \bar{\psi }\left(\bar{z},\bar{t}\right)=\{\begin{array}{cc} \frac{\pi c_{0}}{2T}\cos(\pi \bar{z}/L), & \bar{t}>0,\bar{x}\in W_{0},\\ 0, & \text{otherwise}. \end{array} \end{array}$$ (8)

Using Eq (6) we follow the time development of the particle distribution through all wave-sections.

Eq (6) is clearly an approximate representation for finite sized particles (with partial account through *D*_*th*_) [18]. For particles of finite size, higher order contributions arising from (a) collisions of two or more particles, (b) reduced available volume for diffusion and (c) a modified flow field, would have to be considered. Nevertheless, we expect that zeroth-order behavior (addressing the questions of whether or not there is net transport, the relative influences of diffusion and convection, and the role of recirculation) can be represented by such a description.

The boundary conditions that complement Eq (6) are the conditions of no particle flux through the tube wall and of axi-symmetry, respectively:

$$\begin{array}{c} n\cdot \bar{\nabla }\bar{c}=0,\;\;\;\;\;\bar{x}=\left(\bar{h}\left(\bar{z}\right),\bar{z}\right), \end{array}$$ (9)

and

$$\begin{array}{c} \frac{\partial \bar{c}}{\partial \bar{r}}=0,\;\;\;\;\;\bar{r}=0. \end{array}$$ (10)

Using the same normalisation procedure that led to Eq (3), complemented by the density normalisation $c=\bar{c}/c_{0}$ and time normalisation $t=\bar{t}/T$, the convective diffusion equation, Eq (6), becomes

$$\begin{array}{ccc} \frac{\partial c}{\partial t} & = & -\nabla \cdot \{-\nabla (\alpha c)+\beta cu\}+\psi \\ & = & \alpha [\frac{1}{r}\frac{\partial }{\partial r}(r\frac{\partial c}{\partial r})+\frac{\partial^{2}c}{\partial z^{2}}]-\beta [u_{r}\frac{\partial c}{\partial r}+u_{z}\frac{\partial c}{\partial z}]+\psi , \end{array}$$ (11)

where the dimensionless constants are defined as *α* = *D*_*th*_*T*/*L*^2^ and *β* = (Δ*P*)_*amp*_*T*/*μ*, *i.e.*, ratios of diffusive and convective strengths, respectively, to system-intrinsic values. The ratio *β*: *α* is the mass transfer Peclet number, *p*_*e*_, *viz*, the ratio of convective to molecular mass transfer. The non-dimensional particle generation term in Eq (7) is

$$\begin{array}{c} \psi \left(z,t\right)=\frac{B^{2}}{h^{2}\left(z\right)}[\cos(2\pi z)+1], \end{array}$$ (12)

for *t* ≥ 0, and appears only in one wave-section of the tube and only in the second initial value problem.

In this work we consider the numerical solution of Eq (11) for a range of values of *α* and *β* to ascertain the relative importance of diffusion versus convection for transport in longitudinally asymmetric capillaries, for a range of different tube geometries.

In S1 Appendix (Section C) we complement the analysis and simulation results presented herein with an analogous numerical study adopting the generation function in Eq (8) for *t* > 0 and the simpler initial condition *c* = 1 in *W*_0_. A comparison of the two sets of results will show that the qualitative behavior found is independent of the detailed nature of the chosen $\bar{\psi }\left(\bar{z},\bar{t}\right)$ function and of the initial condition, $\bar{c}\left(\bar{r},\bar{z},0\right)$, although this scenario is different to the scenario of no particle generation.

### Numerical approach

To solve the one-dimensional, simplified version of integral Eq (4) (which is not reproduced here due to its size and complexity), the tube boundary curve over which integrals are performed is partitioned into *N* elements according to a grid defined along the *z*-axis; the integrals are then re-expressed as a sum of integrals over these small elements. For a sufficiently fine grid, the unknowns, which are the surface forces, are assumed constant over the elements and extracted from under the integral signs; the segmented line integrals over the remaining known quantities are then approximately evaluated using the trapezoidal rule. The equations making up this boundary element approximation, together with boundary condition (2), comprise a linear system of 2*N* algebraic equations for the 2*N* unknown components of the force distribution on the tube surface, **f** = (*f*_*r*_, *f*_*z*_). These equations are solved using an International Mathematics and Statistics Library (IMSL) routine in Fortran. More numerical details are given in Islam et al. [1]. Within the creeping flow approximation, given a slowly varying pressure difference, Δ*P*(*t*), the fluid velocity field is assumed to adapt instantaneously to changes in the pressure.

Once the force distribution on the tube surface is known, it is used to calculate the fluid velocity profile in the tube interior via the one dimensional version of Eq (5). It follows from the assumption that the particle distribution does not influence the fluid flow field and the condition of periodicity, that a velocity evaluation at position ${\bar{x}}_{0}$ in the central wave-section is periodically reproduced in all wave-sections at points modulo *L* of the central point, *i.e.*, at points ${\bar{x}}_{k}={\bar{x}}_{0}+kL\hat{z}$ for *k* ∈ *Z*.

The assumption of an instantaneously responsive flow field to a slowly varying pressure difference implies that the time dependence of **u** corresponds to that of Δ*P*(*t*). Defined over a single period only, the non-dimensional pressure differences which we consider here are,

$$\begin{array}{c} \begin{array}{cccc} \Delta P_{a}\left(t\right) & = & P_{0}\sin(2\pi t), & \;0\leq t<1,\\ \Delta P_{b}\left(t\right) & = & P_{0}\cos(2\pi t), & \;0\leq t<1,\\ \Delta P_{c}\left(t\right) & = & -P_{0}\sin(2\pi t), & \;0\leq t<1. \end{array} \end{array}$$

In the above, *P*_0_ ≡ 1 (normalized by (Δ*P*)_amp_) is a fundamental, constant pressure amplitude; the dimensional angular frequency, *ω* = 2*π*/*T*, is prescribed with corresponding period, *T* (with $\bar{t}$ scaled by *T*, the argument of the sinusoidal functions involves only the factor of 2*π*).

In contrast to the method used to solve the fluid dynamic problem, we solve Eq (11), with accompanying boundary and initial conditions, by an explicit discretization scheme applied to a finite number of wave-sections. In the majority of cases, 61 wave-sections in all were considered to be the simulation domain; with *K* = 30 sections on either side of a central section. The spatial domain was partitioned into a rectangular grid ${\left\{\left(z_{i},r_{j}\right)\right\}}_{i,j=1}^{N,M}$ based on uniform linear grids of size Δ*r* and Δ*z*, respectively, established for given tube dimensions, where *N*(*M*) is the number of grid points in the *z*(*r*)−direction in one wave-section. Although it may have been sensible to invoke a nonuniform grid in order to get better resolution near corner regions, we found that if the grid was sufficiently fine, no accuracy issues emerged. Details of the numerical approach can be found in S1 Appendix (Section A).

## Simulation results

Neglecting particle-particle and particle-surface forces, the only physical mechanisms that contribute to the transport of particles through a channel are (Brownian) diffusion and convection. The factors that may influence the extent of transport include the channel shape, the relative geometric dimensions of the channel, fluid viscosity *μ*, particle size *a*, the flow characteristics (specifically, the existence or absence of fluid recirculation and the applied pressure profile) and the applied pressure. The model adopted here takes account of these features to varying degrees of approximation. For example, particle size is indirectly taken into account through its appearance in *D*_*th*_. This level of approximation is consistent with Eq (6).

The two main theoretical questions we address here are, firstly, does net transport occur in channels of nonuniform cross-section and, secondly, what factors do then contribute? A secondary question concerns the influence of the particle generation term itself. Our principal results, used to answer these questions, are expressed in terms of the cross-section and wavelength-averaged partial moments of the particle distribution:

$${\langle c_{n}\rangle }_{k}\left(t\right)=\int_{\left(2k-1\right)/2}^{\left(2k+1\right)/2}\left(\int_{0}^{h\left(z\right)}z^{n}c\left(r,z,t\right)2\pi rdr\right)dz,$$ (13)

for *n* = 0, 1, 2, …, *k* = −*K*, …, *K*.

**Fig 2:**
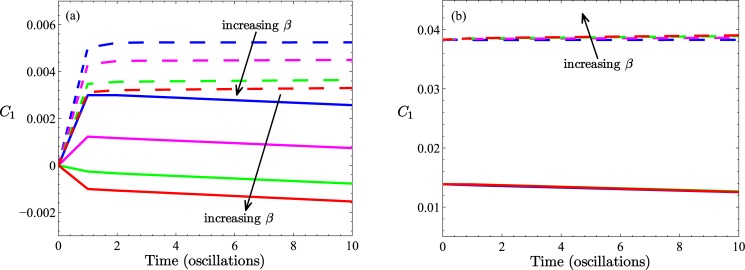
Results of first moment calculations for the first initial value problem of a fixed number of confined particles. Fig (a) is based on initial condition Eq (7) evaluated at *t* = 0, while results in (b) are based on the simpler state $\bar{c}\left(\bar{r},\bar{z},0\right)=c_{0}$ for $\bar{x}\in W_{0}$ and zero otherwise. Two different tube profiles are shown, both with the same throat radius, *B* = 0.2. Solid lines are for *A* = 0.24 (no recirculation), dashed lines are for *A* = 0.48 (recirculation). Results are displayed for *β* = 0, 100, 200, 300; Peclet numbers are *p*_*e*_ = 0, 1000, 2000, 3000 as indicated by arrows. In (b), results for the *A* = 0.24 tube (solid lines) are all but indistinguishable on the scale of the figure. The dependence on *β* is indicated by the arrow. In all cases, simulations were performed with *α* = 0.1 and Δ*P*_*a*_(*t*). The physical significance of positive and negative values of the first moment are schematically represented by the insets to Figs 3(a) and 7(a), respectively.

In particular, the accumulated measure

$$C_{0}\left(t\right)=\overset{K}{\underset{k=-K}{\sum }}{\langle c_{0}\rangle }_{k}\left(t\right),$$ (14)

is used to track the total number of particles in the tube as a function of time. The second important quantity we employ is the time dependent first moment overall:

$$C_{1}\left(t\right)=\overset{K}{\underset{k=-K}{\sum }}{\langle c_{1}\rangle }_{k}\left(t\right),$$ (15)

which is a measure of the asymmetry of the distribution evaluated over the entire simulated tube. For future reference we point out that for the choice of generation function, Eq (7), for both longitudinally symmetric and asymmetric tubes, the initial value, *C*_1_(0), is zero. This is to be contrasted with the case presented in S1 Appendix (Section C) (and shown in Fig 2), in which we consider an alternative initial condition and generation function.

### Transient behavior: No particle generation

Considering the dynamic behavior of a fixed number of particles initially confined to a section of a long tube, it is expected that under diffusive influences alone and for a longitudinally symmetric tube profile (including a straight cylindrical tube) particles would disperse to equal degrees in both directions. Such is not the case for a periodically *asymmetric* tube as illustrated by the two *β* = 0 cases in Fig 2(a) and 2(b); the tube having the larger expansion regions results in a larger distribution asymmetry (larger first moment, *C*_1_(*t*)). In Fig 2(a) the state of the distribution asymmetry is apparently established by *t* = 1 (time point measured in pressure oscillations) and maintained constant thereafter for the tube with the larger expansion regions and decreasing for *t* > 1 for the tube with the smaller expansion regions. As already mentioned the initial particle distribution in (a) was chosen to give a zero first moment regardless of tube shape, which contrasts it with (b) where different shapes (*A* = 0.24 and *A* = 0.48) result in different initial first moments. Since diffusion is governed by concentration gradients it is not surprising to find the different dynamic behavior in Fig 2(a) and 2(b) brought about by the different initial distributions. For the system depicted in Fig 2(a), oscillatory convection effects, superimposed on diffusion, alter the dynamics quantitatively in the tube with the larger expansion regions (which also possess recirculation zones), and qualitatively in the case of the tube with the smaller expansion regions (no recirculation). In the latter case there is clearly a switch in net particle transport (from positive *z*, to negative *z*) which appears for a *β* value between 100 and 200 (Peclet number, 1000 < *p*_*e*_ < 2000). In contrast, for the case of Fig 2(b) there does not appear to be any variation (with *β*) in the first moment at any anniversary of the pressure oscillation.

### Transient behavior: Particle generation in central wave-section

#### Diffusion only: Profile asymmetry as a necessary condition for transport

**Fig 3:**
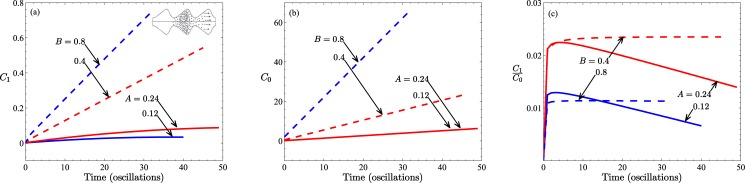
Results for different expansion region to throat radius ratios, *A*/*B* in the case of diffusion only (*β* = 0, *α* = 0.1). Solid lines are for different expansion amplitudes, *A* with fixed throat radius *B* = 0.2, while the dashed lines are for different throat radius, *B* with fixed expansion amplitude *A* = 0.48. The solid and dashed lines with the same colour indicate the same ratio *A*/*B*. (a) Shows the first moment and gives an indication of asymmetric transport, (b) shows the zeroth moment or total mass in the tube and (c) shows the ratio of the two. The inset to (a) depicts schematically the physical significance of positive values of the first moment.

The data depicted in Fig 3 readily answers the first main question of whether it is possible for preferential transport to occur. Fig 3(a)–3(c) show representative results of the first moment, *C*_1_(*t*), of the particle distributions, the total particle numbers, *i.e.*, the zeroth moment *C*_0_(*t*), and the ratio of the two, respectively. Although no convection is yet assumed (*β* = 0) the data is plotted as a function of time measured in units of equivalent cycles of the pressure. The curves in all three panels reflect the response to increases in expansion region (increasing *A*) or increases in the throat region (increasing *B*). The zeroth moments have been normalized by the particle numbers initially contained within the central wave-section. That the *C*_1_ curves do not remain constant over time signifies a disparity between the amount of material transported to the right compared with what has been transported to the left (by diffusion alone). The precise conditions under which the results in Fig 3 are derived are given in the caption. Of particular note is the fact that the direction of net transport is in the direction of the leading edge of the saw tooth profile (see Fig 1(b)).

If we modify the tube shape by displacing laterally the position of the expansion peak to give a symmetric triangular wave-section, while maintaining the same throat width (*B*) and height of expansion peak (*A*), we obtain results (data not shown) for the condition of geometric symmetry. In this special case we find no difference in the amount of material transported to the right or left. That is, we find no net transport of particles: *C*_1_(*t*) ≡ 0 for all *t* ≥ 0. Asymmetry in shape is clearly a critical factor. However, another critical factor is the aspect ratio of the tube profile, *A*/*B*. A decrease in this ratio diminishes the importance of the expansion region, placing greater emphasis on the influence of the throat. However, since this can be achieved in two ways, different consequences ensue for transport (Fig 3(a)–3(c)). As mentioned earlier, with our choice of *ψ* the particles are deliberately distributed unevenly at *t* = 0 to counter the profile asymmetry so as to give a zero first moment initially. Thus, the density of particles is greater in the negative *z*−half of the central wave-section. Given the relatively larger volume available immediately to the right of center, the concentration gradient results in a greater number of particles finding their way to the positive *z*-half of the tube domain.

In the first set of curves of Fig 3 (dashed lines) the height of the expansion zone is kept fixed (*A* = 0.48) for two values of the throat radius, *B*: 0.4 and 0.8. The second set (solid lines) features a constant throat radius (*B* = 0.2) and decreasing expansion zone height: 0.24 and 0.12. These specific combinations of *A* and *B* constrain the geometric ratio *A*/*B* to the common values of 1.2 and 0.6, respectively. A more important reason for this choice will become apparent in a later section. Since the two sets of curves do not superimpose, this ratio is not a universal number, which suggests that different experimental designs will necessarily have different transport characteristics.

Note that decreasing either *B* or *A* decreases the wave-section volume. However, the nature of *ψ* is such that the number of particles contained within the tube decreases only in the former case but remains constant in the latter case (Fig 3(b)). On the other hand, decreasing either *B* or *A* decreases the first moment, *C*_1_(*t*), as the profile adopts a more symmetric shape. Not surprisingly, the zeroth moment (Fig 3(b)) increases with time in all cases as more particles are injected into the system, with the largest increases appearing for the larger systems (*A* = 0.48 and *B* = 0.4, 0.8).

Considering the time development, one of the most important conclusions to draw from (Fig 3(a) and 3(c)) is that, under the simulated conditions, the action of diffusion alone results in a net distribution of particles in the positive *z*-direction, *i.e.*, in the direction of the leading edge of the saw-tooth profile. Taken in conjunction with the qualitatively similar results given in S1 Appendix (Section C), we conclude that the movement towards the positive *z*-direction is not due specifically to our choice of particle generating function, even though *ψ*(*z*, *t*) does influence the quantitative degree of propagation. When the throat is sufficiently narrow, the expansion region has the dominant influence, with the concentration gradient driving more particles in the positive *z*-direction. This influence increases with increasing *A*. Increases in *B* at fixed asymmetric *A* not only increase the total volume in the tube, but also the total particle number generated through *ψ* ($1\geq h_{min}^{2}/h^{2}\left(z\right)\geq B^{2}/{\left(A+B\right)}^{2}\to 1$ for *B* ≫ 1). There will thus be an increasing proportion of particles produced in the leading edge half of the central wave-section with increasing *B*, which will promote a larger degree of diffusion in the positive *z*-direction. As *A* is decreased, the slopes of the first moment profiles decrease in magnitude. In the limit *A* → 0 for fixed *B*, we obtain a straight cylindrical tube for which there is no net direction of transport of particles (*C*_1_ ≡ 0).

Thus, we arrive at our fundamental proposition that tube asymmetry is a necessary condition for net particle transport by the process of diffusion. The associated conclusion, given that we arrive at the same outcome with two choices of generating function (Eq (7) here and Eq (S26) of S1 Appendix (Section C)) is that the qualitative behavior is not governed by the method of introducing particles. It is worth noting that for *t* ≫ 1, the large expansion zone results *A* = 0.48 in Fig 3(c) (with analogous outcomes in the convective case, see later) indicate the linear relationship *C*_1_(*t*) = *κC*_0_(*t*), with the proportionality coefficient, *κ*, being a positive, time-independent, decreasing function of *B*, while for the narrow throat case (*B* = 0.2), the proportionality coefficient is a linear decreasing function of time but increasing function of expansion height.

**Fig 4:**
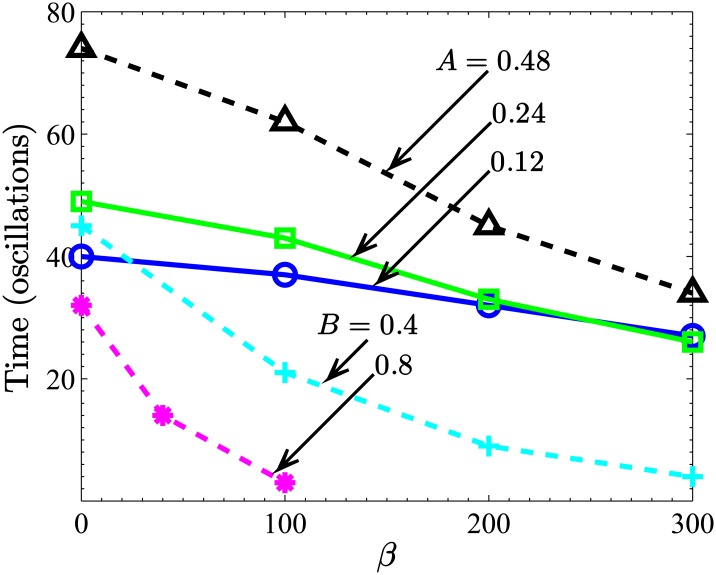
The time at which particles first reach *k* = ±10 (i.e., when the total mass in wave-section *W*_±10_ is greater than 10^−4^) for different tube profiles. The solid lines are for different *A* with fixed *B* = 0.2, while the dashed lines are for different *B* with fixed *A* = 0.48. In all cases *α* = 0.1.

We remark, finally, that the end points of the curves in Fig 3(a) and 3(b) are indicative of the times taken for the particle distributions to reach the 10*^th^* wave-section on one or other side of the central wave-section in our simulated system, *W*_±10_. These are also the ordinate axis intercepts of the curves in Fig 4 (measured in equivalent cycles of a pressure oscillation, *T*). The latter figure summarizes the fact that for constant expansion zone dimensions, the system with the greatest (smallest) throat opening, facilitating (restricting) passage through the tube, has the shortest (longest) transport time. However, it is also apparent that the more accentuated is the expansion zone, the slower the progression of the particles. From a design perspective, these results suggest that the slower progress with an accentuated expansion zone can be offset by a sufficiently large throat. This is discussed further in the next section.

#### Diffusion and convection: Positive or negative reinforcement of transport?

**Fig 5:**
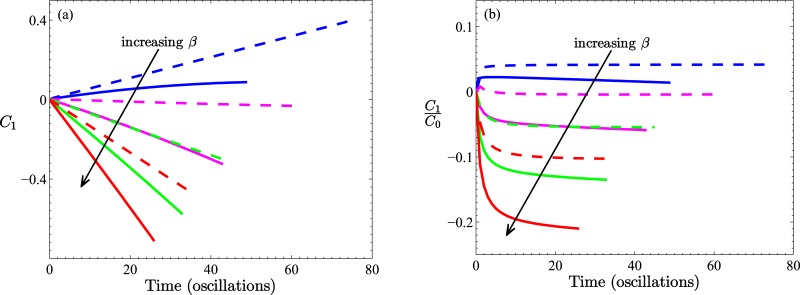
(a) The first moment, and (b) the ratio between the first and zeroth moment. Two different tube profiles are shown, both with the same throat radius, *B* = 0.2. Solid lines are for *A* = 0.24 (no recirculation case), dashed lines are for *A* = 0.48 (recirculation). Results are for various *β* values, *β* = 0, 100, 200, 300 as indicated by arrows; *α* = 0.1 in all cases. In all cases, simulations were performed with Δ*P*_*a*_(*t*).

As the ratio *A*/*B* can be altered either by increasing the throat radius at a fixed expansion dimension or by exaggerating the expansion zone at a fixed throat size, it is not surprising that different convective behavior can result. The different effects are, moreover, compounded by the coupling of convection with diffusion. Fig 5(b) depicts the system’s response (*C*_1_(*t*)/*C*_0_(*t*)) to increasing strength of convection (*β* ≥ 0) for an applied pressure of Δ*P*_*a*_(*t*) = sin(2*πt*) at constant oscillation period. Two profile combinations (*A* = 0.48, *B* = 0.2 with *A*/*B* = 2.4 and *A* = 0.24, *B* = 0.2 with *A*/*B* = 1.2), are considered. In the cases shown, convection biases the particle distribution toward negative *z*-values, the effect of which is reinforced with increased *β*. Although both tube shapes resulted in *positive* first moments in the case of diffusion alone (see Fig 3(a) and 3(c)), with convection present (specifically developing as sin(2*πt*)), a greater proportion of particles now advance in the *negative* *z*-direction. The near-horizontal asymptotes of the ratio of moments (Fig 5(b)) again imply proportional relationships, *C*_1_ = *κC*_0_, this time with *κ* < 0 in all non-zero *β* cases. Since the total particle number *C*_0_ is an increasing function of time as a result of the constant injection of particles (all cases lie on a single curve since *B* is kept constant (data not shown)), the first moment increases in magnitude at a proportional rate (Fig 5(a)). That is, the peak of the particle distribution moves progressively toward *z* = −∞, *i.e.*, in the direction of the profile’s trailing edge. We remark here that we get qualitatively similar behavior (see Fig C of S1 Appendix) using our alternative choice of *ψ*(*z*, *t*), which supports the impression that this trend is independent of how particles are introduced into the tube.

**Fig 6:**
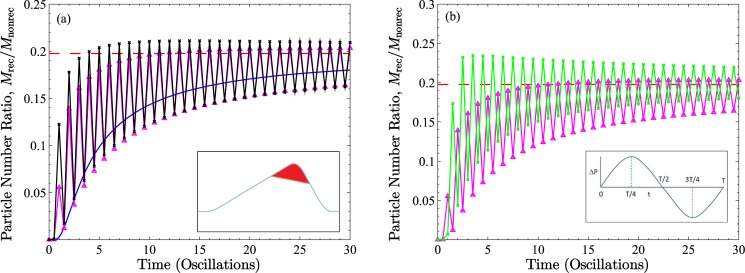
Plots of the time dependent ratios of total numbers of particles found within recirculation regions (depicted in red in inset to figure (a)) of wave-sections *W*_−2_ and *W*_2_ to the total numbers of particles found in the rest of the tube section. The values are results of volume integrals taken over the axisymmetric tube. The geometric conditions are *B* = 0.2 and *A* = 0.48 (which results in fluid recirculation), with transport conditions for (a) of *α* = 0.1 and *β* = 0 (solid blue line), *β* = 200 (pink solid line and triangles) and *β* = 300 (black solid line and crosses). Data points are at quarter periods of the pressure oscillation, $\bar{t}=nT/4$ for *n* = 0, 1, 2, …, although symbols for odd numbered quarter periods have been removed for clarity of presentation. The inset to (b) shows one pressure oscillation with an indication of where data points are taken. In (b) we show the *β* = 200 results for wave-section *k* = 2 (pink solid line and triangles) and wave-section *k* = −2 (green solid line and crosses). For comparison, the red dashed lines depict the ratio of the volumes of the abovementioned regions.

**Fig 7:**
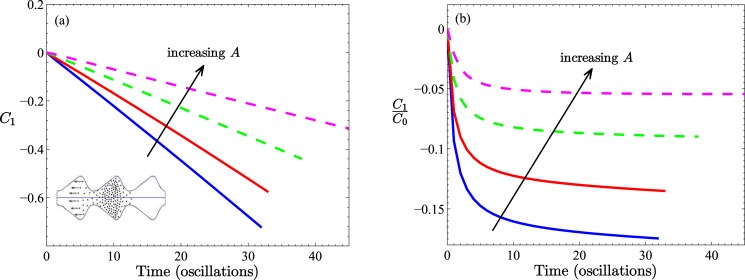
Comparison of results for different expansion amplitudes, *A*, with fixed *B* = 0.2. All results are for *α* = 0.1, *β* = 200. The solid lines are cases with no-recirculation (*A* = 0.12, 0.24), while the dashed lines are for cases with recirculation (*A* = 0.36, 0.48). (a) The first moment and (b) the ratio between the first and zeroth moments. The inset to (a) depicts schematically the physical significance of negative values of the first moment.

**Fig 8:**
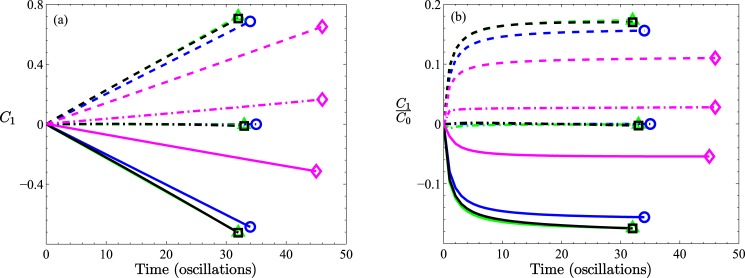
Results for four different tube shapes: Straight (circles), symmetric triangular (*A* = 0.12, triangles), asymmetric saw tooth without recirculation (*A* = 0.12, squares), and asymmetric saw tooth with recirculation (*A* = 0.48, diamonds). In each case *B* = 0.2. Three pressure profiles are shown: sinusoidal, Δ*P*_*a*_(*t*) (solid), negative sinusoidal, Δ*P*_*c*_(*t*) (dashed) and cosine, Δ*P*_*b*_(*t*) (dot-dashed). All curves are plotted for *α* = 0.1, *β* = 200. (a) The first moment and (b) the ratio between the first and zeroth moments.

As to the results themselves, in the case of convection and diffusion, with a pressure differential Δ*P*_*a*_ = sin(2*πt*) driving the fluid initially in the positive direction, the net particle motion is in the negative *z*-direction. The counterintuitive behavior (featured in Figs 5–8) is due to the interplay between the continual addition of particles in the central wave-section and the specific form of the pressure gradient. It can be understood by appeal to the following argument. Consider the simpler system of particles in a straight tube subjected only to convection (*α* = 0), and assume that the fluid displacement amplitude is less than one cell in length. In this case, the sinusoidal flow will first convect the particles initially present, forwards and then backwards, returning them to their initial location after one complete pressure cycle. However, all through this oscillation, particles are being generated in the *W*_0_ wave-section. Thus, in the first half-period of oscillation, particles are present in the zeroth and first wave-sections only. During the second half-period of fluid oscillation, all particles are convected in the negative *z*-direction. At the conclusion of the first pressure oscillation (at *t* = 1) particles will be present in the *W*_0_ *and* the *W*_−1_ wave-sections only. Thus, at *t* = 1, the first time point plotted in the figures, the first moment of the distribution will be negative. The process is repeated during the second and subsequent pressure cycles, with particles continually being added to these two “blocks”. The net result, with more particles specifically and progressively added to wave-section *W*_−1_, steadily shifts the balance of the particle distribution to negative *z*. Including the effect of diffusion only smears this pristine state of a two-wave-section concentration to include particles in neighboring wave-sections, on either side: *k* positive and negative. Similarly, allowing for expansion regions (nonzero *A*) modifies the picture further, but the underlying convective process remains the dominant influence. Some further qualifying comments on this phenomenon appear in the next section.

A summary of the times the particles first reach either wave-section *W*_−10_ or *W*_+10_ as a function of convection strength, *β*, for some different geometric conditions is provided in Fig 4. As already remarked, the greater the throat dimension, the quicker the particles are transported to the ends of the simulated system.

At this point it is worth drawing attention to the different behavior demonstrated by the two transient scenarios (*i.e.*, the case of no particles generated in the tube’s central wave-section versus the case of particles continually added in that wave-section). In absolute terms, the inclusion of particle generation in one wave-section (irrespective of form, see S1 Appendix (Section C)) is sufficient to change the nature of particle transport: compare Figs 2(a) and 3(a) and 5(a). However, in relative terms there is less distinction. Since particles are continually produced in time in this second transient scenario and as we have found *C*_1_ = *κC*_0_ in this scenario, the most appropriate comparison is between the *C*_1_ results shown in Fig 2(a) and the *C*_1_/*C*_0_ results of Fig 3(c), and more clearly those of Fig 5(b).

According to the findings reported in Islam, et al. [1], beyond some critical amplitude, A*, of the expansion region, which depends to varying degrees on other geometric factors (throat width, the degree of asymmetry and width of the expansion region, and somewhat on the degree of smoothing of the profile), the flow field can exhibit one or more zones of recirculation in the expansion regions. It is not unreasonable then for particles that diffuse into one such region to become caught in a recirculation flow, provided the strength of the hydrodynamic flow is sufficient to dominate over diffusive motion. Thus, for sufficiently large *β* there will be regular periods within which trapped particles will circulate in these zones and not propagate from one wave-section to the next. However, for a temporal oscillatory flow field there will always be periods when the velocity field will be “weak” in some sense compared to diffusion, allowing the particles to diffuse out of these zones into or near to the throat region where they can be convected out of that section in the next cycle. The natural question to ask is whether this process assists or hinders the general diffusive movement of particles. That is, the question is whether recirculation opposes or reinforces diffusive transport. A partial answer can already be deduced from the results discussed earlier. The data shown in Fig 5, referring to the convective behavior in tubes whose shape proportions have the ratio *A*/*B* of 1.2 or 2.4, shows that although convection *opposes* the diffusive trend toward positive *z* values, forcing the mean of the distribution to negative *z*, recirculation, which is present in the case *A*/*B* = 2.4, reduces this influence.

Plots of ratios of instantaneous particle numbers in the recirculation regions of wave-sections *W*_−2_ and *W*_2_ to particle numbers in the remainder of those wave-sections are shown in Fig 6. Data points correspond to quarter periods of pressure oscillation (symbols at odd numbered quarter periods have been removed for improved clarity). The smooth continuous curve in Fig 6(a) is the diffusion-only result (*α* = 0.1, *β* = 0), while the two other series of data points refer to convective diffusion conditions of *α* = 0.1, and *β* = 200 and *β* = 300. For comparison we include (as red dashed lines) the value of the ratio of the volumes of the two regions concerned (*V*_*rec*_/*V*_*nonrec*_ = 0.1976). Wave-sections *k* = −2 and *k* = 2 were chosen somewhat judiciously to ensure sufficient advance to steady state conditions within a reasonable number of oscillations, yet be representative of what would transpire in all sections. In (a) the ratio maxima appear at completions of pressure oscillations, while ratio minima appear during pressure lulls midway through oscillations. With time there is an obvious increasing trend toward steady state both for the *β* = 0 case as well as for the (common) bottom envelope through points of minima for the nonzero *β* values. The (different) upper envelopes through the points of maxima appear to plateau to constant values sooner than do the minima. It is not clear from the figure but the upper envelopes actually have a slight negative slope with time. As a whole, the results suggest the tendency to converge toward the asymptotic value given by the volume ratio, which is consistent with the picture of a uniform distribution at steady state, to be discussed shortly. In (b) the dynamic situations on either side of the central wave-section are contrasted; most obvious is the 180° phase difference between the results, which is not surprising given the oscillatory nature of the pressure. But what is also clear is the particle distribution asymmetry between *k* = 2 and *k* = −2 underlying the net negative first moment shown in Fig 5.

Complementary information is found in the results shown in Fig 7 where we compare transport in tubes with a common throat dimension, *B* = 0.2, but with subcritical and supercritical expansion zone dimensions. These results reinforce the idea that recirculation reduces the tendency of convection to transport particles in the negative *z*-direction; particles become trapped in regions of recirculation flow and are thus unavailable for convective transport during a significant proportion of a pressure cycle. Recirculation is thus an important design characteristic to consider in the fabrication of micro- and nano-channels. Moreover, within the class of non-recirculation flows, a factor of two difference in expansion amplitude does not result in a proportionate change in net transport. On the other hand, within the class of recirculation flows, a 50% change in expansion zone amplitude results in a two fold change in the first moment (Fig 7(a)). Note again that since the throat dimension is kept constant at *B* = 0.2, the total number of particles generated increases linearly with time but is independent of *A* (data not shown).

Finally, in Fig 8 results of the somewhat elementary consideration of different time dependent pressure gradients, Δ*P*_*a*_(*t*), Δ*P*_*b*_(*t*) and Δ*P*_*c*_(*t*) are presented. With both mechanisms of diffusion and convection acting within either a symmetric (triangular) or an asymmetric (saw-tooth) tube, one would expect a non-zero mean distribution of particles, with the direction of bias being determined by the sign of the pressure gradient. Naturally, the total particle count does not display any dependence on pressure profile (data not shown), which is a feature that can be utilized to check on the numerics. By construction, both symmetric and asymmetric profiles have zero ordinate intercepts (*C*_1_(0)) in Fig 8(a) and 8(b). However, for pressure oscillations of Δ*P*_*a*_(*t*) and Δ*P*_*c*_(*t*), all cases immediately depart from zero thereafter.

According to our earlier theoretical explanation, with Δ*P*_*c*_(*t*) the convective field couples with diffusion to promote propagation toward positive *z*, while with Δ*P*_*a*_(*t*) the convective field opposes diffusion to result in propagation toward negative *z*. Although this is captured in the results shown in Fig 8(a) and 8(b), the all but reflective symmetry about the time axis suggests that for the cases of low (*A*/*B* = 0.6) or no (*A*/*B* = 0) expansion region, for which no recirculation zones appear, diffusion does not play a decisive role, even though it remains an active participant, spreading the particles along the tube (the simulations are terminated when the particles have reached wave-section *W*_±10_, which indicates that diffusion is still important). It is interesting that compared with the case of a straight cylindrical tube, the presence of an expansion region, whether symmetrically positioned or asymmetrically positioned, enhances the transport slightly (particles reach *W*_±10_ sooner). However, for the case of a significant expansion region, *A*/*B* = 2.4, for which recirculation zones appear, the reflective symmetry is broken, suggestive of a more significant cooperation between diffusion and recirculation in the manner described previously, biasing the transport in the direction of the leading edge of the saw-tooth tube (positive *z*-direction), but delaying transport somewhat (the lines for these cases are terminated at larger *T* values, indicating that particles reach *W*_±10_ later).

From an experimental perspective, it is significant that both Δ*P*_*a*_(*t*) and Δ*P*_*c*_(*t*) are experimentally reasonable temporal functions. The influence of Δ*P*_*b*_(*t*) is midway between the other two, with *C*_1_(*t*) being very close to zero for the symmetric tube systems and even for the low *A*/*B* = 0.6 case of an asymmetric tube. For the asymmetric tube with an exaggerated expansion region, *A*/*B* = 2.4, the cosine time dependent pressure amplitude is still midway between the other two, but non-zero. The results for the alternative initial state and generating function (data not shown) are qualitatively consistent indicating a lack of dependence on the form of function assumed.

### Initial state of a uniform particle distribution

We imagine that particle generation has persisted sufficiently long or that conditions have been so manufactured that the tube has become filled with particles to a uniform concentration (*c* = 1) prior to the application of fluid flow. The system in this uniform condition is assumed the initial state for subsequent convective diffusion calculations; the system and its dynamics are then periodic in space, at all times. Consequently, the following conditions of periodicity apply at any location, *z*_0_, along the tube:

$$\{\begin{array}{l} c\left(r,z_{0},t\right)=c\left(r,z_{0}+L,t\right),\\ \nabla c{|}_{z_{0}}=\nabla c{|}_{z_{0}+L}. \end{array}$$ (16)

It is intuitive that when the tube is then subjected to an applied oscillatory hydraulic pressure wave, there will not be any change in the total number of particles in any wave-section (S1 Appendix (Section B)). Superimposing a symmetry argument one would then conclude that for a straight tube as well as for a periodic tube of longitudinally symmetric profile there is no net particle flux in either direction. It does not necessarily follow, however, that no net transport of particles occurs in one or other direction for a periodic tube with an asymmetric profile. To address this question we have undertaken a simulation study of the latter case using, as quantitative measure, the time-averaged particle flux through the throat cross-section at *z*_0_ = 1/2,

$$\begin{array}{ccc} \hat{J} & = & \int_{0}^{1}J\left(t;1/2\right)dt\\ & = & \int_{0}^{1}\int_{0}^{b}\{\beta cu_{z}\sin\left(2\pi t\right)-\alpha \frac{\partial c}{\partial z}\}2\pi rdrdt \end{array}$$ (17)

For all asymmetric profile cases considered, varying the strength of convective strength (*β*), the strength of diffusion (*α*) and the amplitude of the expansion region in the profile (*A*, to capture the cases of recirculation and non-recirculation), the time averaged particle flux through the throat cross-section was zero. This means that not only is the total particle number within each wave-section conserved over a period of sinusoidal pressure oscillation (what exits on the right, enters on the left), at this level of approximation there is also *no net movement of particles in either direction* when averaged over a period of oscillation; what exits on the right during one half of an oscillation, enters again from the right during the other half. Consequently, only under the transient situations considered in and prior to achieving steady state is there net particle movement.

Naturally, this conclusion is based on the model we have employed. It may therefore need revising should an alternative model, that includes higher order particle size effects or nonisotropic diffusion [18], be employed.

## Discussion

Our numerical findings show that different transport characteristics are possible depending on the system characteristics. For the reader’s convenience we summarize our findings in point form.

1. For the transient case tracking the evolution of an initial distribution of a finite number of particles, the initial distribution plays an important role. Distributed to counterbalance the tube shape, the particles are transported in the direction of the leading edge, predominantly by diffusion. Convection reduces the degree to which this occurs and can redirect the transport in the direction of the trailing edge.
2. For the transient case of an initial distribution supplemented by a steady supply of particles in one wave-section:
  - With straight cylindrical and longitudinally symmetric tubes:
    - an oscillatory convective flow field alone will *not* result in net transport;
    - diffusion alone will *not* result in net transport;
    - net transport *is* possible, even in straight cylindrical tubes, when *both* an oscillatory convective flow field and diffusion act. However, for cylindrical tubes and periodic tubes of shallow expansion regions, net transport is dominated by convection.
  - With longitudinally asymmetric tubes:
    - diffusion alone *will* result in net transport, in a direction that is dependent on the geometry of the tube, *i.e.*, on both the relative and absolute dimensions of the expansion and throat portions. In our set up, the direction of net transport is in the direction of the leading edge of the saw tooth;
    - regardless of the direction of diffusive particle transport, the superposition of an oscillatory convective flow field driven by a positive (negative) sinusoidal pressure gradient will convect the particles in the direction of the trailing (leading) edge of the tube, opposing (supporting) the flow direction established by diffusion;
    - diffusion couples with flow recirculation in highly distended, expansion regions of a tube of a given throat radius to modify the degree of transport relative to the case of no recirculation in favor of the direction preferred by diffusion.
  - In the case of both asymmetric and symmetric tube profiles:
    - when both diffusion and convection act, the direction of net transport is strongly dependent on the oscillatory pressure gradient driving the fluid flow.
3. For a tube uniformly filled with particles, the model adopted here predicts that particle number per wave-section is conserved and, moreover, that there is no net transport on average over one period of pressure oscillation.

Our results are arguably conditional on the specific conditions of our simulations, namely tube geometry, initial distribution of particles and finally on the assumed generation of particles in the central wave-section. Although we have demonstrated throughout that qualitative behavior is not dependent on the details of either the initial particle distribution (initial condition) or how particles are introduced in the central section (the generating function)—compare Figs 3–8 and Figs B–D of S1 Appendix—it is reasonable to ask whether the observed qualitative behavior itself is predicated on the existence of any generating source of particles.

In Fig 2 we presented results of calculations of the first moment *C*_1_(*t*) for the case of no particle generating function, with particles initially distributed in two ways, giving rise to either a zero initial first moment 2(a) or a nonzero first moment 2(b). For tubes with small expansion regions, the convection-free case has the particles diffusing out of *W*_0_ according to Eq (6) in such a way that the first moment decreases linearly (after the first oscillation). In this situation, without a continual source of particles to influence behavior, diffusion subsequently drives more particles in the negative *z*-direction. The effect of a superimposed convection (under a positive sinusoidal pressure difference, Δ*P*_*a*_(*t*)) is to shift this trend, in the physical manner discussed earlier, and under sufficiently high flow rates to establish a net negative first moment from the outset. For tubes with large expansion regions, particles diffuse to the right creating a net positive first moment, which is again counteracted, but to a lesser extent, by fluid convection (compare differences between dashed lines and solid lines in Fig 2(a)).

We have not investigated the first initial value problem for any greater number of oscillations due to limited numerical accuracy (dispersed particle concentrations and moment calculations quickly become comparable to the numerical error). Nevertheless, one can reasonably conclude that, while the particular details of a source function *ψ*(*z*, *t*) may not matter qualitatively, the existence of a continual source of particles is itself influential in determining net transport behavior. The follow-up question to ask is: which scenario, with its accompanying physical response, is the most relevant? Presuming that to achieve particle transport, some form of particle reservoir is required, it would seem more appropriate to consider the condition of a source of particles, represented here by *ψ*(*z*, *t*), and the response shown in Figs 3–8. This is certainly the more relevant case to drug infusion in the vascular system [13, 14].

In view of our findings, we conclude that there is a strong dependence on experimental design, not only regarding the shape and dimensions of the tubes.

## Concluding remarks

Particle transport through macroscopic vessels can occur under the action of gravity (sedimentation) [19, 20], of an applied electric field (electrophoresis) [21, 22] or of a fluid flow (convection) [23]. We have investigated particle transport in longitudinally asymmetric capillaries assuming the action of both diffusive and convective mechanisms. We have undertaken a study primarily of the influence of tube geometry on magnitude, direction and rate of transport of particles in axi-symmetric tubes of saw-tooth shape. Assuming a physical model of forced diffusion where the convective element, an underlying fluid velocity field, is assumed unaffected by the presence of the particles, we find a range of transport outcomes depending explicitly on tube geometry and applied pressure profile. In particular, we have considered the effect of replacing a pressure gradient that is a simple sine function of time with a negative sine and a cosine time dependent pressure gradient, with important consequences on the direction of preferred transport. One area still to be explored is the effect of the relative differences between characteristic times of particle diffusion, particle convection and temporal oscillation of the pressure gradient. This consideration is with the view to a more detailed study of the influence of recirculation flow in the expansion regions of the tube. In a future publication we hope to report on this aspect, as well as a more direct comparison with experimental results.

## Supporting information

S1 Appendix Analysis details and supplementary results. (PDF)
