# Supplementary material for: Convective and diffusive effects on particle transport in asymmetric periodic capillaries
Source: PLoS One. 2017 Aug 25;12(8):e0183127. doi: 10.1371/journal.pone.0183127 (PMC5571904; doi:10.1371/journal.pone.0183127)
Supplement: S1 Appendix — (PDF) [file pone.0183127.s001.pdf]

Convective and diffusive effects on particle transport in asymmetric periodic capillaries

Nazmul Islam, Stanley J Miklavcic, Bronwyn H Bradshaw-Hajek, Lee R. White.

S1 Appendix

A. Numerical solution of the convective-diffusive equation

We solve the non-dimensional convection-diffusion equation Eq. (11), with initial and boundary conditions, by the explicit finite difference method (FDM). The finite difference scheme is based on the forward difference representation for the time derivative and the central difference formula for spatial derivatives. Let Δz and Δr represent the 1D grid spacing in the z and r directions, respectively, and let Δt denote the time increment. Let $N(M)$ be the number of grid points in the $z(r)$-direction.

To address the problem of the boundary of the tube not being coincident with the spatial grid, we extend the grid beyond the physical tube boundary and identify two sets of points. The first, denoted Γ, is the discrete set of points where the physical boundary intersects the two-dimensional grid. The second, denoted C, is the set of interior points that are closest to the physical boundary. The numerical scheme, which is outlined in the sections below, then involves solving the governing system of equations on three sets of grid points, the set Γ, the set C and the set of all remaining interior points. These sets are depicted in Figure A.

Concentrations at interior points

Finite difference formulae are used in place of continuous derivatives in (11), which is then rewritten to solve for the discretized concentration $c_{\mathrm{i,j}}^{s+1}$ at interior spatial grid position (i, j) (black grid points in Figure A), at time $s + 1$.

By Taylor's theorem, we have

$$c_{\mathrm{i,j}}^{s+1} = c_{\mathrm{i,j}}^{s} + \Delta t \frac{\partial c}{\partial t} + \frac{(\Delta t)^2}{2!} \frac{\partial^2 c}{\partial t^2} + ..., \tag{S1}$$

where all derivatives are evaluated at the grid-point (i, j), at time s. Rewriting Eq. (S1)

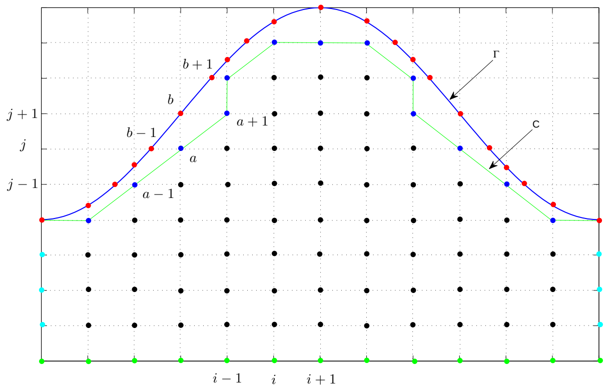

Figure A. (Color online) Finite difference space grid, which differentiates the three sets of discrete points: the set Γ (red points on blue curve which intersects grid lines), the set C (blue points on piecewise linear green curve) and the set of all remaining interior points (black points at grid vertices). Note that the Γ points can either be points of intersection of vertical or horizontal grid lines. Green points indicate the centerline of the tube, while light blue points are points on the edges of a spatial period of the infinite periodic tube.

we deduce the forward difference approximation for the time derivative,

$$\frac{\partial c}{\partial t} = \frac{c^{s+1}_{\rm i,j} - c^{s}_{\rm i,j}}{\Delta t} + O[(\Delta t)]. \tag{S2}$$

Similarly, the Taylor's series for $c^s_{\rm i\pm1,j}$ evaluated about the central value $c^s_{\rm i,j}$ is given by

$$c^s_{\rm i\pm1,j} = c^s_{\rm i,j} \pm \Delta z \frac{\partial c}{\partial z} + \frac{(\Delta z)^2}{2!}\frac{\partial^2 c}{\partial z^2} \pm .... \tag{S3}$$

Solving for $\dfrac{\partial c}{\partial z}$ and $\dfrac{\partial^2 c}{\partial z^2}$ from the latter two equations, we obtain the central difference approximations,

$$\frac{\partial c}{\partial z} = \frac{c^s_{\rm i+1,j} - c^s_{\rm i-1,j}}{2\Delta z} + O[(\Delta z)^2], \tag{S4}$$

$$\frac{\partial^2 c}{\partial z^2} = \frac{c^s_{\rm i+1,j} - 2c^s_{\rm i,j} + c^s_{\rm i-1,j}}{(\Delta z)^2} + O[(\Delta z)^2]. \tag{S5}$$

Analogously, we obtain the finite difference approximations for the radial derivatives,

$$\frac{\partial c}{\partial r} = \frac{c^s_{\rm i,j+1} - c^s_{\rm i,j-1}}{(\Delta r)^2} + O[(\Delta r)^2], \tag{S6}$$

$$\frac{\partial^2 c}{\partial r^2} = \frac{c^s_{\rm i,j+1} - 2c^s_{\rm i,j} + c^s_{\rm i,j-1}}{(\Delta r)^2} + O[(\Delta r)^2]. \tag{S7}$$

Substituting equations (S2) and (S4) - (S7) into equation (S1) and rewriting, we deduce the concentration value $c_{\mathrm{i,j}}^{s+1}$ at the (i,j) nodal point at time point $(s+1)$,

$$\begin{aligned} c_{\mathrm{i,j}}^{s+1} = c_{\mathrm{i,j}}^{s} \quad &+ \quad \alpha\Delta t\left[\frac{c_{\mathrm{i+1,j}}^{s} - 2c_{\mathrm{i,j}}^{s} + c_{\mathrm{i-1,j}}^{s}}{(\Delta z)^2} + \frac{c_{\mathrm{i,j+1}}^{s} - 2c_{\mathrm{i,j}}^{s} + c_{\mathrm{i,j-1}}^{s}}{(\Delta r)^2} + \frac{c_{\mathrm{i,j+1}}^{s} - c_{\mathrm{i,j-1}}^{s}}{2r_{\mathrm{j}}\Delta r}\right] \\ &- \quad \beta\Delta t\left[u_{\mathrm{z}}\frac{c_{\mathrm{i+1,j}}^{s} - c_{\mathrm{i-1,j}}^{s}}{2\Delta z} + u_{\mathrm{r}}\frac{c_{\mathrm{i,j+1}}^{s} - c_{\mathrm{i,j-1}}^{s}}{2\Delta r}\right] + \Delta t\psi_{\mathrm{i}}^{s}. \end{aligned} \tag{S8}$$

To proceed further and evaluate the updated concentration values at all interior points at successive time steps using Eq. (S8), requires updating the concentration values at the point sets, C and Γ. To calculate these concentrations, it turns out to be useful to adopt a different labeling convention for these point sets. Points on C were indexed by the variable $a \in [1, N_a]$ while points on Γ were indexed with the variable $b \in [1, N_b]$, with both counted from the point in the center of the throat. In general, $N_b > N_a$, usually greater by a factor of two. Note that the physical boundary can intersect the grid by either intersecting the horizontal grid lines or intersecting the vertical grid lines. The two cases require subtly different approaches.

Concentrations along contour C

To update the points on the piecewise contour, C, we invoke a non-uniform grid in both z and r. Keeping the first three terms in Newton's interpolation equation [1], the z-derivatives for $a \in C$ points that are adjacent to Γ points corresponding to horizontal grid-line intersections, can be approximated by

$$\begin{aligned} c'(z_{\mathrm{a}}) \quad &= \quad \frac{c_b - c_a}{\lambda\Delta z} - \frac{c_{\mathrm{b}} - (\lambda+1)c_{\mathrm{a}} + \lambda c_{\mathrm{i}}}{\lambda(\lambda+1)\Delta z} \\ &\quad - \frac{2c_{\mathrm{a}} - (\lambda+1)(\lambda+2)c_{\mathrm{a}} + 2\lambda(\lambda+2)c_{\mathrm{i}} - \lambda(\lambda+1)c_{\mathrm{i-1}}}{2(\lambda+1)(\lambda+2)\Delta z}, \\ c''(z_{\mathrm{a}}) \quad &= \quad \frac{c_{\mathrm{b}} - (\lambda+1)c_{\mathrm{a}} + \lambda c_{\mathrm{i}}}{\lambda(1+\lambda)\Delta z^2} \\ &\quad + \frac{(1-\lambda)[2c_{\mathrm{b}} - (\lambda+1)(\lambda+2)c_{\mathrm{a}} + 2\lambda(\lambda+2)c_{\mathrm{i}} - \lambda(\lambda+1)c_{\mathrm{i+1}}]}{2\lambda(\lambda+1)(\lambda+2)\Delta z^2}, \end{aligned} \tag{S9}$$

where $z_{\mathrm{a}} - z_{\mathrm{i}} = \Delta z$, $z_{\mathrm{b}} - z_{\mathrm{a}} = \lambda\Delta z$ for $0 < \lambda < 1$. The r-derivatives are approximated using Eqs. (S6) and (S7).

Similarly, r-derivatives for $a \in C$ points that are adjacent to Γ points corresponding

to vertical grid-line intersections can be approximated by

$$\begin{aligned}
c'(r_\mathrm{a}) &= \frac{c_\mathrm{b}-c_\mathrm{a}}{\gamma\Delta r} - \frac{c_\mathrm{b}-(\gamma+1)c_\mathrm{a}+\gamma c_\mathrm{j}}{\gamma(\gamma+1)\Delta r} \\
&\quad - \frac{2c_\mathrm{a}-(\gamma+1)(\gamma+2)c_\mathrm{a}+2\gamma(\gamma+2)c_\mathrm{j}-\gamma(\gamma+1)c_\mathrm{j-1}}{2(\gamma+1)(\gamma+2)\Delta r}, \\
c''(r_\mathrm{a}) &= \frac{c_\mathrm{b}-(\gamma+1)c_\mathrm{a}+\gamma c_\mathrm{j}}{\gamma(1+\gamma)\Delta r^2} \\
&\quad + \frac{(1-\gamma)[2c_\mathrm{b}-(\gamma+1)(\gamma+2)c_\mathrm{a}+2\gamma(\gamma+2)c_\mathrm{i}-\gamma(\gamma+1)c_\mathrm{j+1}]}{2\gamma(\gamma+1)(\gamma+2)\Delta r^2},
\end{aligned} \tag{S10}$$

where $r_\mathrm{a}-r_\mathrm{j}=\Delta r$, $r_\mathrm{b}-r_\mathrm{a}=\gamma\Delta r$ for $0<\gamma<1$. The z-derivatives are approximated using Eqs. (S4) and (S5). For each point $a \in C$, the i, j and b indices referred to in Eqs. (S9) and (S10) are uniquely identified.

Using these finite difference approximations in Eq. (11), concentration values at the time step $s+1$, for points in the contour set C, are updated using the above information provided at time step s,

$$\begin{aligned}
c_\mathrm{a}^{s+1} &= c_\mathrm{a}^{s} + \alpha\Delta t\left[c''(z_\mathrm{a}) + \frac{c'(r_\mathrm{a})}{r_\mathrm{a}} + c''(r_\mathrm{a})\right]^s \\
&\quad -\beta\Delta t\left[u_\mathrm{z}c'(z_\mathrm{a}) + u_\mathrm{r}c'(r_\mathrm{a})\right]^s + \Delta t\psi_\mathrm{a}^s.
\end{aligned} \tag{S11}$$

We have found that the additional terms in the discrete approximation to the first partial derivatives are necessary to obtain a comparable level of numerical accuracy to the discrete second derivatives.

Concentration calculation along boundary Γ

Having identified, for each point b in Γ, the closest a and $a+1$ points on contour C, we can derive leading order approximations to $\left.\frac{\partial c}{\partial z}\right|_\mathrm{b}$ and $\left.\frac{\partial c}{\partial r}\right|_\mathrm{b}$ in terms of the components of the unit normal to the surface boundary. The leading order Taylor approximations [1] to the concentrations at a and $a+1$ are

$$c(a) = c(b) + (z_\mathrm{a}-z_\mathrm{b})\left.\frac{\partial c}{\partial z}\right|_\mathrm{b} + (r_\mathrm{a}-r_\mathrm{b})\left.\frac{\partial c}{\partial r}\right|_\mathrm{b}, \tag{S12}$$

$$\begin{aligned}
c(a+1) &= c(b) + (z_\mathrm{a+1}-z_\mathrm{b})\left.\frac{\partial c}{\partial z}\right|_\mathrm{b} \\
&\quad +(r_\mathrm{a+1}-r_\mathrm{b})\left.\frac{\partial c}{\partial r}\right|_\mathrm{b},
\end{aligned} \tag{S13}$$

while the no-flux condition (the non-dimensional version of Eq. (9)) is given by

$$n_{\mathrm{z}}|_{\mathrm{b}} \left.\frac{\partial c}{\partial z}\right|_{\mathrm{b}} + n_{\mathrm{r}}|_{\mathrm{b}} \left.\frac{\partial c}{\partial r}\right|_{\mathrm{b}} = 0, \tag{S14}$$

Solving for $\left.\frac{\partial c}{\partial z}\right|_{\mathrm{b}}$ and $\left.\frac{\partial c}{\partial r}\right|_{\mathrm{b}}$ from equations (S12), (S13) and (S14) we obtain

$$\left.\frac{\partial c}{\partial z}\right|_{\mathrm{b}} = \frac{n_{\mathrm{r}}|_{\mathrm{b}}[c(a) - c(a+1)]}{n_{\mathrm{r}}|_{\mathrm{b}}(z_{\mathrm{a}} - z_{\mathrm{a+1}}) - n_{\mathrm{z}}|_{\mathrm{b}}(r_{\mathrm{a}} - r_{\mathrm{a+1}})}, \tag{S15}$$

$$\left.\frac{\partial c}{\partial r}\right|_{\mathrm{b}} = \frac{n_{\mathrm{z}}|_{\mathrm{b}}[c(a) - c(a+1)]}{n_{\mathrm{r}}|_{\mathrm{b}}(z_{\mathrm{a}} - z_{\mathrm{a+1}}) - n_{\mathrm{z}}|_{\mathrm{b}}(r_{\mathrm{a}} - r_{\mathrm{a+1}})}. \tag{S16}$$

The concentrations on the boundary Γ can thus be deduced using either equation (S12) or (S13).

The concentrations at grid points along the tube axis $r = 0$ are updated using the symmetry condition (10), which takes the finite difference form

$$c_{\mathrm{i,1}}^{s+1} = c_{\mathrm{i,2}}^{s+1} \quad \text{for} \quad 2 \leq i \leq N. \tag{S17}$$

The above finite difference method in two spatial dimensions is numerically stable and convergent provided

$$\Delta t \leq \frac{(\Delta z)^2 + (\Delta r)^2}{8\alpha}, \tag{S18}$$

where α is the non-dimensional diffusion coefficient.

B. Steady state transport in a fully periodic system

The objective is to determine the net amount of material, $\Delta\Lambda$, that is transported at steady state out of an arbitrary wavesection of a periodic capillary over one period of pressure. No particle generation is now considered at steady state. We do not specify the nature of the profile asymmetry. Let V denote the interior domain of this wavesection with boundary $S = S_1 \cup S_2 \cup S_3$, where S_1 and S_3 are the planar regions interior to the capillary connecting this wavesection to its near neighbor sections, and S_2 is the solid wall of the tube. Let $\mathbf{n}_i$ be the outward normal to S_i for $i = 1, 2, 3$. The total number of particles present in V at any point in time, t is $\Lambda(t) = \int_V c(\mathbf{x}, t) dV$.

Since V is fixed, the rate of change of Λ with time is

$$\frac{d\Lambda}{dt} = \int_V \frac{\partial c}{\partial t}(\mathbf{x}, t)\ dV. \tag{S19}$$

Replacing the time derivative of c with the right-hand side of the convective-diffusive equation we have

$$\frac{d\Lambda}{dt} = \int_V \{D\nabla \cdot \nabla c - \nabla \cdot (c\mathbf{u}) + c\nabla \cdot \mathbf{u}\}\ dV. \tag{S20}$$

By Gauss' divergence theorem, the first two volume integrals can be rewritten as integrals over the bounding surface:

$$\frac{d\Lambda}{dt} = \int_S \{D\nabla c \cdot \mathbf{n} - c\mathbf{u} \cdot \mathbf{n}\}\, dS + \int_V c\nabla \cdot \mathbf{u}\ dV. \tag{S21}$$

Assuming that the fluid is incompressible, the continuity equation implies that $\nabla \cdot \mathbf{u} = 0$. Consequently, the last volume integral vanishes. Splitting the two remaining surface integrals into integrals over the individual surfaces S_i, respectively, gives

$$\int_S \nabla c \cdot \mathbf{n} dS = \int_{S_1} \nabla c \cdot \mathbf{n}_1 dS + \int_{S_2} \nabla c \cdot \mathbf{n}_2 dS + \int_{S_3} \nabla c \cdot \mathbf{n}_3\ dS \tag{S22}$$

and an analogous partitioning of the integral of $c\mathbf{u}$. The resulting expression for the rate of change of particle number simplifies upon invoking the no particle flux and the stick boundary conditions on S_2 and noting that $\mathbf{n}_1 = -\widehat{\mathbf{z}} = -\mathbf{n}_3$, where $\widehat{\mathbf{z}}$ is the unit vector in the positive z direction. The surface integrals over S_2 vanish and the rate of change of Λ reduces to

$$\frac{d\Lambda}{dt} = D\int_{S_1} \left(-\frac{\partial c}{\partial z}\right) dS \quad + \quad D\int_{S_3} \left(\frac{\partial c}{\partial z}\right) dS \tag{S23}$$

$$- \quad \int_{S_1} (-u_z c) dS - \int_{S_3} (u_z c) dS. \tag{S24}$$

However, at steady-state and for strict spatial periodicity between wavesections, $c(\mathbf{x} \in S_1, t) = c(\mathbf{x} \in S_3, t)$ and $\mathbf{u}(\mathbf{x} \in S_1, t) = \mathbf{u}(\mathbf{x} \in S_3, t)$. Consequently, $d\Lambda/dt \equiv 0$ and we see that averaging over one period of pressure oscillation produces no net particle transport: $\Delta\Lambda = 0$. Note that this result is independent of tube profile within

the wavesection. The only constraint is of periodicity. Thus, only when the system is still in a transient state can there be change in the total number of particles in any wavesection of a periodic capillary.

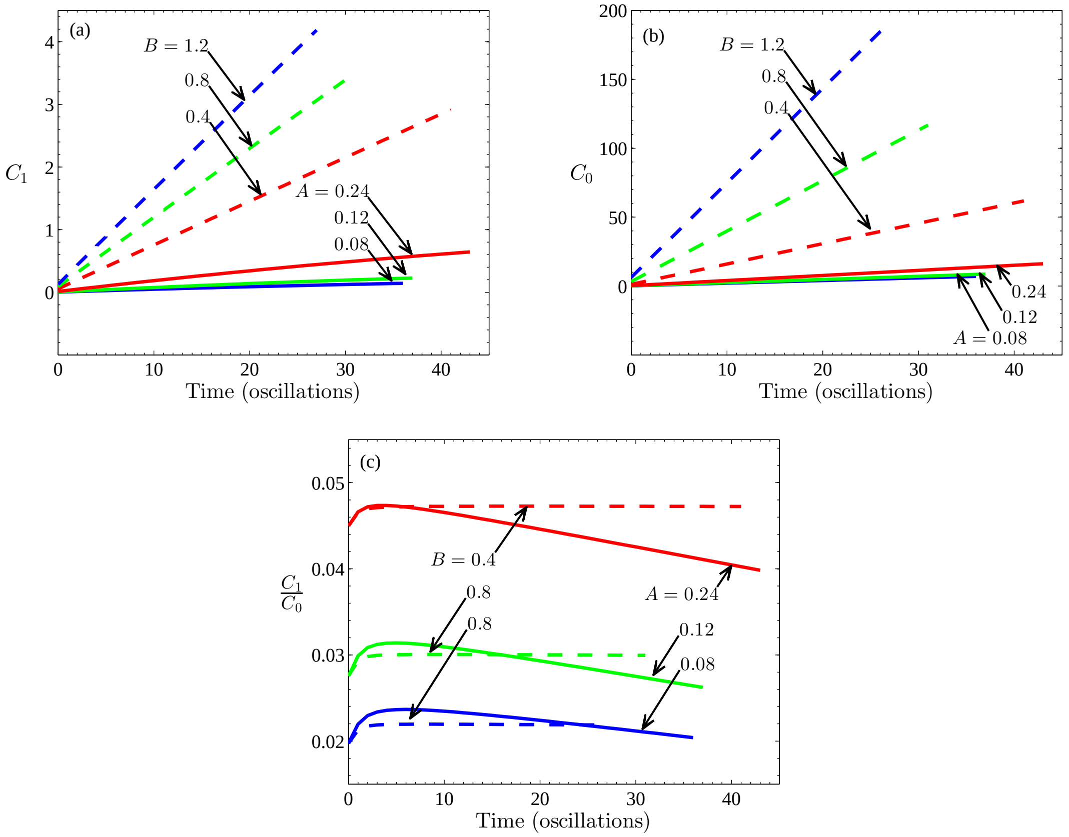

Figure B. (Color online) Results for different expansion region to throat radius ratios, A/B in the case of diffusion only ($\beta = 0,\ \alpha = 0.1$). Solid lines are for different expansion amplitudes, A with fixed throat radius $B = 0.2$, while the dashed lines are for different throat radius, B with fixed expansion amplitude $A = 0.48$. The solid and dashed lines with the same colour indicate the same ratio A/B. (a) Shows the first moment and gives an indication of asymmetric transport, (b) shows the zeroth moment or total mass in the tube and (c) shows the ratio between the two.

C. Initial value problem with initial uniform particle distribution

To address the question of whether the qualitative character of the results presented in the main body of the paper are dependent on the choice of generating function, $\bar{\psi}(\bar{z}, \bar{t})$, and initial condition, we performed an analogous series of simulations using the simpler

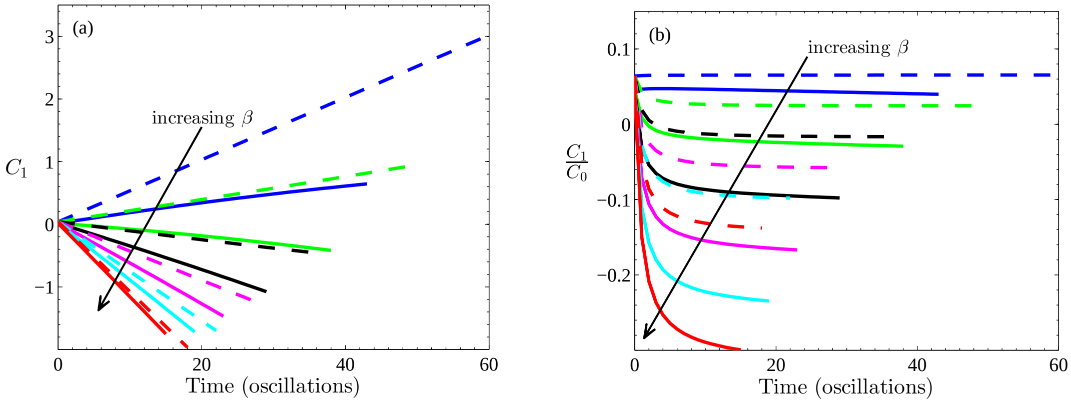

Figure C. (Color online) (a) The first moment, and (b) the ratio between the first and zeroth moment. Two different tube profiles are shown, both with the same throat radius, $B = 0.2$. Solid lines are for $A = 0.24$ (no recirculation case), dashed lines are for $A = 0.48$ (recirculation). Results are for various β values, $\beta = 0,\ 100,\ 200,\ 300,\ 400,\ 500$; $\alpha = 0.1$ in all cases. In (b), the longest (blue) line in each tube profile indicates the lowest beta value, $\beta = 0$, while the shortest (red) line in each case indicates the highest beta value, $\beta = 500$.

initial state

$$\bar{c}(\bar{r}, \bar{z}, 0) = \begin{cases} c_0, & \bar{\mathbf{x}} \in W_0, \\ 0, & \text{otherwise}, \end{cases} \tag{S25}$$

where $W_0 = \left\{(\bar{r}, \bar{z}); \bar{r} \in (0, \bar{h}(\bar{z})), -L/2 \leq \bar{z} \leq L/2\right\}$ is the central wavesection, and using a generation function of the form

$$\bar{\psi}(\bar{z}, \bar{t}) = \begin{cases} \dfrac{\pi c_0}{2T}\cos(\pi \bar{z}/L), & \bar{t} > 0, \bar{\mathbf{x}} \in W_0, \\ 0, & \text{otherwise}. \end{cases} \tag{S26}$$

As in the case of Eq. (7) of the main body of the paper, both of the above conditions assume a constant, uniform (in r) supply of particles within the central wave-section. However, in contrast to Eq. (7), the initial condition is uniform in z as well as in r, while the generating function, although smoothly approaching zero at the ends of the central wave-section, is independent of the shape of the tube profile. Both conditions result in non-zero first (positive) moments, and lead to different values of zeroth and first order moments with changing tube dimensions. The results for this choice of functions are presented here. All other geometric and dynamic conditions have been kept identical to those adopted in the main paper. Our numerical findings below are qualitatively consistent with the results presented in the body of the paper. This

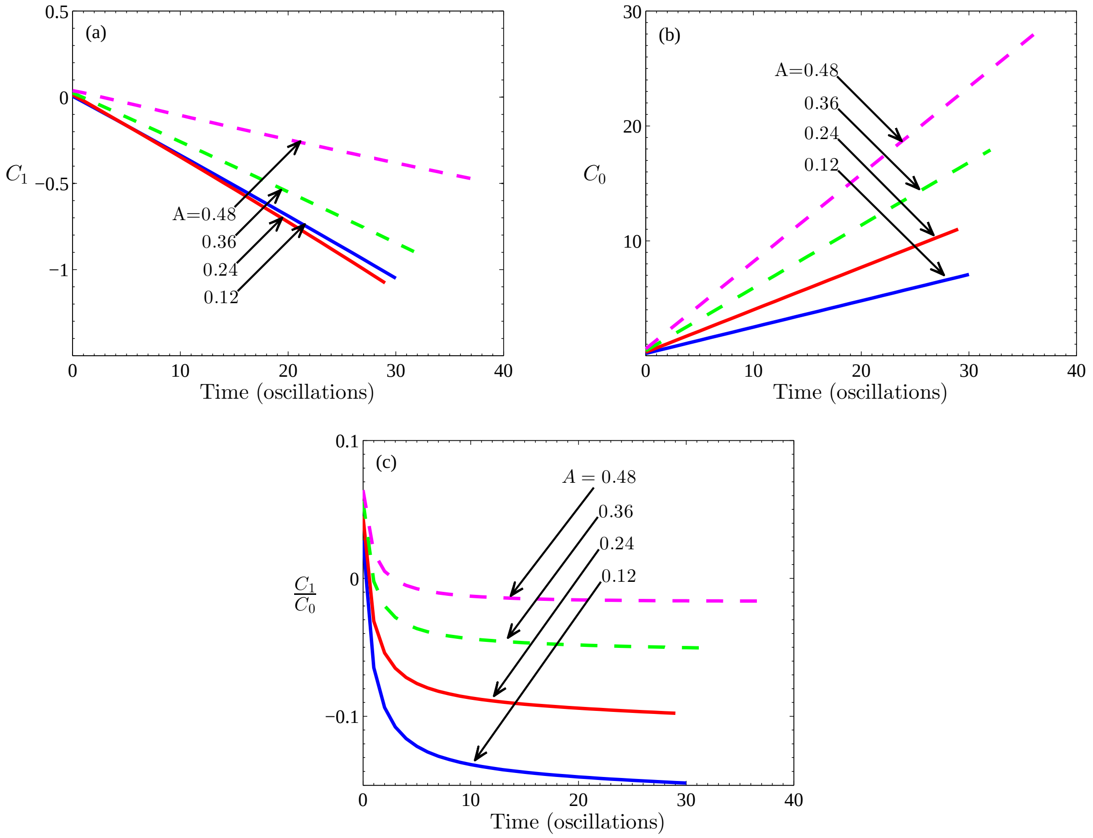

Figure D. (Color online) Comparison of results for different expansion amplitudes, A, with fixed $B = 0.2$. All results are for $\alpha = 0.1$, $\beta = 200$. The solid lines are cases with no-recirculation, while the dashed lines are for cases with recirculation. (a) The first moment, (b) the zeroth moment, and (c) the ratio between the two.

supports the conclusion that the qualitative trends for particle transport are independent of the nature in which particles are introduced into the tube and governed only principally by the tube geometry. That said, the quantitative level of net transport is influenced by the generating function.

References

1. H. Jeffreys and B Jeffreys, Methods of Mathematical Physics 3rd Ed., Cambridge University Press, Cambridge, 1956.
